# *Enterococcus gallinarum* Spontaneous Bacterial Peritonitis in an HCV Cirrhotic

**DOI:** 10.1155/2015/898235

**Published:** 2015-05-07

**Authors:** Hussein Abidali, Maheen Sheikh, Moustapha Abidali, Ali Abidali, Hamoudi S. Farraji, Andrew C. Berry

**Affiliations:** ^1^Department of Medicine, Banner Good Samaritan Medical Center, Phoenix, AZ 85006, USA; ^2^Department of Medicine, Division of Gastroenterology, Banner Good Samaritan Medical Center, Phoenix, AZ 85006, USA; ^3^Midwestern University, Glendale, AZ 85308, USA; ^4^Department of Medicine, University of South Alabama, Mobile, AL 36688, USA

## Abstract

We present the case of a 60-year-old Caucasian male with history of hepatitis C viral cirrhosis with portosystemic encephalopathy and ascites with evidence of spontaneous bacterial peritonitis (SBP) with absolute neutrophil count (ANC) of 944 cells/*µ*L blood. Despite adequate treatment, the abdominal pain and elevated creatinine continued to persist. Initial ascites fluid cultures returned back positive for growth of *Enterococcus gallinarum*. Empiric antibiotics were then substituted with ampicillin/sulbactam. Our case of *Enterococcus gallinarum* causing SBP is only the seventh case reported in the literature to date.

## 1. Introduction

The most common and life-threatening infection in decompensated cirrhosis is spontaneous bacterial peritonitis (SBP), with prevalence of 10%–35% and in hospital mortality rates ranging from 20 to 40% [1]. The diagnosis of SBP is based on positive ascitic fluid bacterial culture and an ascitic fluid absolute polymorphonuclear leukocyte count ≥250 cells/mm [1]. Based on American Association for the Study of Liver Diseases (AASLD) guidelines, empirical third-generation cephalosporins remain the treatment of choice for suspected SBP as it covers 95% of the flora including the three most common isolates:* Escherichia coli*,* Klebsiella pneumoniae*, and* Streptococcal pneumonia* [2]. Although these three are the most common isolates responsible for SBP, other unusual organisms should be considered in immunosuppressed patients, particularly in case of significant animal exposure.

## 2. Case Report

**Figure 1:**
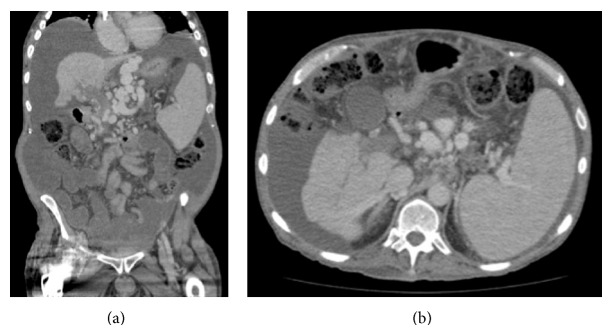
(a) CT abdomen/pelvis with and without contrast in coronal portal phase showing complete occlusion of the entire portal vein at the level of the confluence of the superior mesenteric vein and mild cavernous transformation of the main portal vein. Splenomegaly noted, measuring 19.6 cm in craniocaudal dimension with moderate to large ascites. (b) CT abdomen/pelvis with and without contrast in axial portal phase showing a cirrhotic liver with no arterial hyperenhancing hepatic lesion or lesion with washout. There are multiple calcifications in the main pancreatic duct at the neck resulting in dilatation of the pancreatic duct involving the body and tail measuring up to 1.3 cm.

A 60-year-old Caucasian male with history of hepatitis C viral cirrhosis with portosystemic encephalopathy and ascites was admitted after routine therapeutic paracentesis (9.2 L removed) revealed evidence of spontaneous bacterial peritonitis (SBP) with absolute neutrophil count (ANC) of 944 cells/*µ*L blood. The patient complained of mild generalized abdominal pain without fever, chills, nausea, vomiting, chest pain, or dyspnea. His past medical history includes successful treatment of chronic hepatitis c virus with evidence of sustained virological response on admission given negative HCV PCR viral loads. The patient lives on a small farm and reveals frequent animal exposure to rabbits, squirrels, and dogs along with oral-to-oral contact through kissing all his pets. He denied any consumption of alcohol. The patient presented with recurrent ascites refractory to step one diuretics with furosemide 40 mg daily and spironolactone 100 mg daily. He was hospitalized three months prior for hepatic encephalopathy and ascites with no clear precipitant identified at that time. Laboratory results from three months ago were notable for creatinine of 1.05 and an overall MELD of 8. The patient struggled with issues of ascites (requiring almost weekly taps) and portosystemic encephalopathy since that time. Computed tomography (CT) of the abdomen and pelvis with and without contrast revealed ascites, splenomegaly, and cirrhosis with portal venous hypertension and new-onset complete occlusion of the portal vein at the level of the confluence of the superior mesenteric vein (Figures 1(a) and 1(b)). Initial vitals were all within normal limits including temperature, heart rate, blood pressure, respiratory rate, and oxygen saturation. On admission, the patient had a Child-Pugh Score of 9, Class B, and a MELD of 19. The following laboratory results were obtained:

- WBC 5.3 × 103/*µ*L;
- Hgb 9.3 g/dL;
- platelets 49 × 103/*µ*L;
- total bilirubin 1.4 mg/dL;
- INR 1.4;
- albumin 3.2 ng/dL;
- creatinine 2.11 mg/dL;
- BUN 78 mg/dL.

Antibiotic therapy with ceftriaxone was initiated and a follow-up paracentesis was performed revealing an ANC decline from 944 to 579 cells/*µ*L blood. Despite adequate treatment, the abdominal pain and elevated creatinine continued to persist. Initial ascites fluid cultures were pending and eventually returned back positive for growth of* Enterococcus gallinarum*. Antibiotic susceptibilities to* E. gallinarum* cultured from ascites fluid showed antibiotic susceptibilities to penicillin, linezolid, and vancomycin. Blood cultures drawn on admission were negative. Empiric antibiotics were then appropriately substituted with ampicillin/sulbactam and continued for 5 days with resultant ascites fluid showing resolution of SBP with negative follow-up ascites cultures. His abnormal kidney function tests on admissions were of new onset. Given his continued decline of kidney function, triple therapy for hepatorenal syndrome (HRS) was initiated on day 3 of hospitalization including albumin human (albumin human 25%), midodrine, and octreotide. The patient's kidney function and urine output continued to decline despite HRS therapy and eventually required initiation of hemodialysis 1 day after the resolution of his SBP and then continued to require HD indefinitely.

## 3. Discussion

Our case of* Enterococcus gallinarum* causing SBP in a 60-year-old Caucasian man with hepatitis C viral cirrhosis is only the seventh case reported in literature to date [3–5]. Despite adequate treatment with empiric antibiotics, our patient continued to experience abdominal pain with persistent elevated creatinine. The initial choice of empiric antibiotics for treating SBP is very broad and should take into account local resistance patterns and recent antibiotic use, but repeat paracentesis should be performed if the ascitic fluid analysis or response to treatment is atypical. Emergence of SBP due to* Enterococci* has been rising in the last decade resulting in treatment failures and poorer outcomes. Specifically,* Enterococcus gallinarum* is not known to cause human disease. It is suspected to have colonized the intestinal tract and have been acquired orally from prior exposure to animals [4]. Infections caused by more unusual organisms affecting the intestinal tract have been noted in alcoholic cirrhotic patients; however, our patient did not consume alcohol [4, 5].* E. gallinarum* strains have a specific vanC gene that confers low-level vancomycin resistance [6]. Colonization is common in patients with severe disease in the intensive care unit setting and associated with previous carbapenem use and nephropathy [7]. However, vanC vancomycin resistance enterococci are not commonly transmitted between patients and do not warrant contact precautions [3], just proper antibiotic coverage. Only six prior cases of* Enterococcus gallinarum* spontaneous bacterial peritonitis have been reported to date, with only one previously reported in a patient with direct animal exposure [3–5].

## 4. Conclusion

Our case represents the importance of getting a thorough patient history, as it is necessary to highlight certain at-risk patient populations for enterococci infection, such as patients with significant animal exposure or those who are immunosuppressed. Since empirical third-generation cephalosporin therapy does not cover* Enterococci*, it is imperative to follow-up cultures and address these patient populations so appropriate antibiotics, such as ampicillin or gentamycin, can be swiftly initiated.
